# Dengue haemorrhagic fever presenting with cholestatic hepatitis: two case reports and a review of literature

**DOI:** 10.1186/1756-0500-7-568

**Published:** 2014-08-26

**Authors:** Jevon Yudhishdran, Rayno Navinan, Asoka Ratnatilaka, Sivakumar Jeyalakshmy

**Affiliations:** Department of Medicine (ward 56), National Hospital of Sri Lanka, Regent street, Colombo, Sri Lanka; Department of Medicine, National Hospital of Sri Lanka, Regent street, Colombo, Sri Lanka; Department of medicine, Colombo South Teaching Hospital, Colombo, Sri Lanka

**Keywords:** Dengue fever, Hepatitis, Cholestasis, Cholestatic jaundice

## Abstract

**Background:**

Dengue fever is a common mosquito borne viral fever in South Asia, which causes significant morbidity and mortality. Dengue fever is well known to involve the liver, especially in dengue hemorrhagic fever. The hepatic involvement is usually that of a mild hepatitis with transaminase derangement without jaundice. In cases of dengue hemorrhagic fever where shock has ensued, a severe hepatitis with gross derangements of transaminases and bilirubin may occur. These are two rare cases of adult patients with dengue hemorrhagic fever presenting with a cholestatic type of jaundice.

**Case presentation:**

This case report describes two female patients aged 30 and 46 years who presented with fever, icterus and biochemical analysis revealed cholestatic jaundice. Evolution of the clinical picture and dropping platelets prompted serological investigations in the form of dengue non-structural protein 1 antigen and dengue immunoglobulin M which confirmed acute dengue infection.

**Conclusion:**

These cases highlight the importance of considering dengue fever as a differential diagnosis even in the presence of a cholestatic jaundice, especially in countries where dengue fever is endemic, and in travelers returning from dengue endemic countries. The early diagnosis of dengue fever and timely institution of supportive fluid management is essential to prevent morbidity and mortality.

## Background

Dengue fever is the commonest mosquito born viral illness known to man, with cases exceeding 2.2 million in 2010 [1]. Dengue viruses (DENV) are classified into four serotypes (DENV-1, 2, 3 and 4). Infection may be clinically asymptomatic or give rise to an undifferentiated fever, dengue fever (DF), dengue haemorrhagic fever (DHF) or dengue shock syndrome (DSS) [2]. One third of the world population is at risk and with outbreaks occurring in cyclical epidemics in Asia, Sri Lanka is at a greater risk as it is a country naturally endemic to dengue [2–4]. An increasing number of patients whom present with the clinical picture of fever, myalgia and arthralgia, can evolve into dengue hemorrhagic fever with plasma leakage, which if not properly managed could very well progress to worsening organ involvement with dengue shock. Liver involvement in dengue fever is typically that of a hepatitis which ranges from mild derangement of liver enzymes to fulminant hepatitis, with manifestations ranging from hypochondrial-pain, hepatomegaly and rarely even jaundice [5]. We describe two patients who had in addition to fever had cholestatic jaundice as an initial presenting symptom which was due to dengue infection.

## Case presentation

### 1st case vignette

A 30 year old South-Asian female presented with 5 day history of high fever associated with severe myalgia, headache and yellowish discoloration of the skin. There was no history of contact with muddy water, alcohol consumption, or use of any drugs including antibiotics, anti-pyretics (acetaminophen) or antiemetics. Furthermore history of blood transfusion, sexual promiscuity or any other high risk behavior could not be elicited. She denied any pale stools or pruritus. At the time of admission, general examination revealed she was febrile and icteric but had no conjunctival hemorrhage, pallor, lymphadenopathy, rashes or scratch marks. The respiratory system was unremarkable and the abdominal examination did not elicit right hypochondrial tenderness, palpable gall bladder or abdominal guarding. The cardiovascular examination was normal and she had an initial blood pressure of 90 mmHg systole and 60 mmHg diastole, without a postural drop. She was conscious, alert, well oriented and nervous system examination was otherwise unremarkable.

The initial complete blood count revealed thrombocytopenia with a platelet count of 69 × 10^9^/L (150–450), a normal white blood cell count (WBC) of 10.4 × 10^9^/L (4–11) with a predominance of lymphocytes (55%) and a hemoglobin of 11.0 g/dL (11–17) . The blood picture mirrored the whole blood analysis and showed only thrombocytopenia, there was no neutrophil left shift or toxic granules and the red cell indices were normal. Liver functions showed a mildly elevated aspartate transaminase(AST) and alanine transaminase(ALT) which were 80 U/L(0–40) and 38 U/L (0–35 ) respectively with an elevated alkaline phosphatase(ALP) value of 450 U/L (100–360). Serum direct and total bilirubin levels were elevated at 6 mg/dL (0–0.3) and 9.1 mg/dL (0.3-1.9) respectively. Serum albumin was found to be reduced with a value of 2.3 g/dL (3.5 -5.5), with a normal international normalized ratio (INR). The renal functions were within normal reference range. The C- reactive protein (CRP) and erythrocyte sedimentation rate (ESR) were also within normal parameters. The following day the platelet count continued to drop and her jaundice deepened. A positive dengue non- structural protein1antigen test (NS1) and a positive dengue IgM antibody ELISA test on day 6 confirmed the clinical suspicion and diagnosis of dengue fever. Hepatitis B surface antigen, hepatitis C antibody, hepatitis A and E antibodies, Ebstein Barr Virus antibody and a retroviral screening were all negative. Leptospira microscopic agglutination test (Lepto MAT) titres were insignificant at 1:200 on day 6 and the titre value remained unchanged when the repeat test was performed on day 12 of the illness (a four -fold rise in titre or a high titre >1:800 is the gold standard for diagnosis). Blood cultures remained repeatedly negative.

The platelet count continued to drop rapidly in the following days reaching the lowest nadir value of 3 × 10^9^/L (150–450) with rising alkaline phosphatase and bilirubin levels (Table 1). On day 6 of the illness the patient became afebrile. As per the local guidelines and WHO dengue guidelines on management of critical phase of dengue hemorrhagic fever, she was maintained on a fluid regimen tailored to her body weight, with strict monitoring of the blood pressure, pulse pressure, heart rate and urine output.

**Table 1:** **Chronology of platelet, PCV and liver biochemistry of both patients in case study 1 and 2**

Day	AST U/L (<34)		ALT U/L (<40)		ALP U/L (100–360)		T.Bilirubin (0.1 -1.2 mg/dL)		D.Bilirubin (<0.3 mg/dL)		Platelet ×10 ^9^/L		PCV	
	Pt 1	Pt 2	Pt 1	Pt 2	Pt 1	Pt 2	Pt 1	Pt 2	Pt 1	Pt 2	Pt 1	Pt 2	Pt 1	Pt 2
5	80		38		450		9.1		6		69		31.1%	
6	100	242	52	120	490	400	10.6	23	7.8	12	30	10	32%	34%
8	116	230	56	98	598	412	12.6	22	9.6	6	3	5	35%	34.2%
9	88	160	50	83	448	625	11.6	17.7	8.8	6.2	20	17	33%	30.1%
10		127		78		631		18		8		31		29.9%
11	70		44		368		4.8		3.0		55	62	32%	29.9%
12		88		45		378		16		3		128		30.8%
13	66		40		300		2		1.4		108		32%	
15	60		40		260		0.8		0.2		166		32%	

(Day- day of the illness, AST- aspartate transaminase, ALT- alanine transaminase, ALP-alkaline phosphatase, T.Bilirubin- total bilirubin, D.Bilirubin- direct bilirubin, PCV-packed cell volume, Pt 1- Patient discussed in case vignette 1, Pt 2- Patient discussed in case vignette 2).

On day 7 the patient became mildly tachypnoeic, and examination revealed reduced air entry in the right lung base which was stony dull to percussion and deep icterus, further abdominal examination elicited a small shifting dullness, signifying ascites. An ultrasound scan was done and confirmed a small right sided pleural effusion, an enlarged liver of 16 cm, without intra or extra hepatic bile duct dilatation, a thickened gall bladder wall of 7 mm in the absence of any gall stones and a sonographic Murphy’s sign could not be elicited. Mild ascites was also noted. The blood pressure and urine output were maintained. A packed cell volume was done and showed only a mild rise. The fluid regimen was continued as per guidance, giving adequate fluids to maintain blood pressure and urine output.

On day 9 of the illness, the patient’s clinical status and jaundice improved. Serial complete blood counts showed rising platelet counts. Similarly liver function tests revealed normalizing alkaline phosphatase and bilirubin levels. The patient was discharged on the 15th day of illness with normal cell counts and liver function parameters.

### 2nd case vignette

A 46 year old previously well South-Asian female, presented with six days of high fever, myalgia and jaundice. She too denied any history of contact with muddy water, recent drug usage, blood transfusion or any high risk behavior. On examination, she appeared ill, febrile to touch and deeply jaundiced. Abdominal examination revealed a mildly tender and firm hepatomegaly. The gallbladder was not palpable nor was there guarding or a positive Murphy’s sign. The respiratory system examination revealed reduced air entry on auscultation and stony dullness to percussion in the base of the right lung. The cardiovascular examination was unremarkable, with a blood pressure of 100mmHg systole and 70 mmHg without a postural drop. The nervous system examination was normal.

Whole blood analysis on presentation demonstrated thrombocytopenia with a platelet count of 10 × 10^9^/L (150–450) and a lymphocytic predominant (51%) white cell count of 7.8 × 10^9^/L (4–11) with a haemoglobin value of 11.6 g/dl with a packed cell volume of 34%. The liver enzymes were elevated with the AST more than the ALT which were 242 U/L (0–40) and 120 U/L(0–35) respectively. The alkaline phosphatase was mildly elevated with a value of 400 U/L (100 – 360), with a high direct bilirubin fraction of 12 μmol/L (0–3.4) and total bilirubin of 23 μmol/L (5–21). Albumin was found mildly reduced at 31 g/L (36–48). The INR was normal. The renal functions were normal. The ultrasound scan of the abdomen and chest confirmed an enlarged liver, with a cranio-caudal length of 16 cm. The gallbladder wall was thickened and measured 6 mm, a negative sonographic Murphy’s sign was noted and no gall stones were visualized. Leakage was confirmed as there was mild ascites and a pleural effusion in the right hemithorax. The ESR was 19 mm for the first hour and the CRP was mildly elevated with a value of 7 mg/L (0.0 – 5.0). The dengue IgM antibody was positive. A Lepto MAT was performed twice on day 7 and day 11 of the illness, and showed a titre of 1:400 (a four -fold rise in titre or a high titre >1:800 is the gold standard for diagnosis) without further rise. A hepatitis screen was performed and was found negative. EBV antibody was also negative. This patient was similarly managed with a fluid regimen tailored to her body weight as per guidance, with strict monitoring of hemodynamic parameters and urine output.

The platelet count continued to drop reaching a nadir of 5 × 10^9^/L (150–450) on day 8 of the illness, and subsequently began to rise. The alkaline phosphatase and bilirubin continued to rise till day 10 of the illness, after which the liver functions began to normalize (Table 1). The patient was discharged on day 12 of the illness, being afebrile for 4 days with a platelet count of 128 × 10^9^/L (150–450) and a near normal bilirubin level.

## Discussion

Dengue infection is usually associated with liver involvement, and this is more frequently seen among women, patients with a primary infection and in those with DHF [5]. This claim is reinforced as hepatomegaly is frequent and commoner in patients with DHF than in those with DF [6, 7] as are transaminase levels higher in DHF/DSS than in DF, though the rise is transient and tend to normalize on recovery [6, 8]. In dengue infections, elevation in serum AST is greater than ALT levels, similar to the pattern seen with alcoholic hepatitis [9]. Though other indicators of liver function such as serum bilirubin and alkaline phosphatase can also be deranged their incidence is relatively less in comparison to the often seen rise in transaminase levels (7.2%, 16.3% and 93.3% respectfully) [10]. The causative dengue serotype is a also a factor with a greater degree of hepatic involvement in infections with DENV-3 and DENV-4 [11]. Furthermore there is a suggested correlation between acetaminophen administration for fever, and the degree of liver damage [12].

The exact pathogenesis of liver injury in dengue is yet to be determined. The liver cells may be damaged through one or more of the following mechanisms: direct cytopathic effect of the virus, killing of virus-infected cells by the host immune response- the activated lymphocytes, antibodies against dengue proteins cross-reacting with host cell proteins and as a nonspecific effect of shock [13, 14]. Another form of hepatic involvement which is known to occur in dengue fever is an acalculous cholecystitis. However these patients will have the characteristic right hypochondrial tenderness and a positive Murphy’s sign with the ultrasound confirming the thickened gallbladder and elicitation of a sonographic Murphy’s sign as well. Furthermore elevations in total bilirubin and alkaline phosphatase are not common in cholecystitis as the inflammation is restricted to the gall bladder [15, 16]. Jaundice is seen in fulminant hepatitis secondary to dengue [17], but neither of our patients progressed to this stage of hepatic dysfunction. The development of bilirubinemia and cholestatic jaundice, without fulminant hepatitis is thought to occur due to ongoing inflammatory process of dengue infection, which can effectively reduce the diameter of the lumen of the biliary canaliculus, causing obstruction. This can be attributed directly or indirectly to the effects of the dengue virus [5].

Alternate causes of cholestatic jaundice should be considered. Ultra-sonographic imaging in our patients failed to demonstrate gall stones, and furthermore the normalizing bilirubin with the recession of jaundice made a sinister cause like carcinoma of the head of the pancreas unlikely. Intestinal flukes such as Fasciola Hepatica may cause an obstructive pattern of jaundice, but the clinical picture seen in acute/liver phase of the infection is different from the characteristic symptoms and biochemical derangement to that of dengue, additionally it is the chronic/biliary phase that classically produces jaundice in Fasciola Hepatica infection [18]. Though exclusion would have been useful, jaundice was unlikely to be attributable to a liver fluke. Our patients had jaundice, but neither of them complained of preceding or associated pruritus. Though known to occur, the frequency and intensity of pruritus has poor correlation to the severity of jaundice, and is more commoner in chronic cholestatic jaundice than in obstructive variant, and even less so when the obstructive pathology is benign . Pruritus can also be absent [19]. Pale colored clay stools, another feature of obstructive jaundice was also absent, and this most likely due to that fact the obstruction was partial. Serological diagnosis of dengue was confirmed using dengue IgM and NS1 as they were positive. Though NS1 is commonly an early indicator it can be detectable even late in the disease and though not the ideal choice, it may be useful even in late presenters considering availability of investigational facilities [20, 21]. Overall the clinical picture, serological diagnosis and rapid improvement of jaundice upon clinical recovery favored cholestatic jaundice being secondary to dengue infection in both our patients.

Cholestatic jaundice in otherwise uncomplicated DF/DHF has been reported only scarcely in medical literature, once in a neonate with dengue fever [22] and two other adults [5]. We report this rare phenomenon in two cases of adult patients with dengue haemorrhagic fever. Further studies are required into the incidence and exact mechanism of hepatic damage giving rise to the observed clinical picture of cholestasis in dengue haemorrhagic fever, which is probably a poorly appreciated hepatic manifestation.

## Conclusion

These cases highlight that in addition to deranged liver enzymes, cholestatic pattern of jaundice may be an under recognized clinical manifestation of dengue fever. A high suspicion of dengue should be present when other supportive clinical and laboratory criteria are present in addition to that of jaundice. Countries that are endemic to dengue and regions that have epidemic outbreaks should be vary of the different presentations that may mask dengue fever.

## Consent

Written informed consent was obtained from both patients for publication of this Case Report. Copies of the written consent are available for review by the Editor-in-Chief of this journal.

## References

[1] **Dengue and severe dengue-Fact sheet N°117**. [http://www.who.int/mediacentre/factsheets/fs117/en/]

[2] Gibbons RV, Vaughn DW (2002). Dengue: an escalating problem. BMJ.

[3] Sirisena PD, Noordeen F (2014). Evolution of dengue in Sri Lanka-changes in the virus, vector, and climate. Int J Infect Dis.

[4] Ooi EE, Gubler DJ (2009). Dengue in Southeast Asia: epidemiological characteristics and strategic challenges in disease prevention. Cad Saude Publica.

[5] de Souza LJ, Nogueira RM, Soares LC, Soares CE, Ribas BF, Alves FP, Vieira FR, Pessanha FE (2007). The impact of dengue on liver function as evaluated by aminotransferase levels. Braz J Infect Dis.

[6] Wahid SF, Sanusi S, Zawawi MM, Ali RA (2000). A comparison of the pattern of liver involvement in dengue hemorrhagic fever with classic dengue fever. Southeast Asian J Trop Med Public Health.

[7] Shah GS, Islam S, Das BK (2006). Clinical and laboratory profile of dengue infection in children. Kathmandu Univ Med J (KUMJ).

[8] Mohan B, Patwari AK, Anand VK (2000). Hepatic dysfunction in childhood dengue infection. J Trop Pediatr.

[9] Chongsrisawat V, Hutagalung Y, Poovorawan Y (2009). Liver function test results and outcomes in children with acute liver failure due to dengue infection. Southeast Asian J Trop Med Public Health.

[10] Kuo CH, Tai DI, Chang-Chien CS, Lan CK, Chiou SS, Liaw YF (1992). Liver biochemical tests and dengue fever. Am J Trop Med Hyg.

[11] Kalayanarooj S, Nimmannitya S (2003). Clinical presentations of dengue hemorrhagic fever in infants compared to children. J Med Assoc Thai.

[12] Clayton TA, Lindon JC, Cloarec O, Antti H, Charuel C, Hanton G, Provost JP, Le Net JL, Baker D, Walley RJ, Everett JR, Nicholson JK (2006). Pharmaco-metabonomic phenotyping and personalized drug treatment. Nature.

[13] Guzman MG, Kouri G (2002). Dengue: an update. Lancet Infect Dis.

[14] Smith DR, Khakpoor A (2009). Involvement of the liver in dengue infections. Dengue Bull.

[15] Sharma N, Mahi S, Bhalla A, Singh V, Varma S, Ratho RK (2006). Dengue fever related acalculous cholecystitis in a North Indian tertiary care hospital. J Gastroenterol Hepatol.

[16] Bhatty S, Shaikh NA, Fatima M, Sumbhuani AK (2009). Acute acalculous cholecystitis in dengue fever. J Pak Med Assoc.

[17] Roy A, Sarkar D, Chakraborty S, Chaudhuri J, Ghosh P (2013). Profile of hepatic involvement by dengue virus in dengue infected children. N Am J Med Sci.

[18] Moghadami M, Mardani M (2008). Fasciola hepatica: a cause of obstructive jaundice in an elderly man from Iran. Saudi J Gastroenterol.

[19] Kremer AE, Oude Elferink RP, Beuers U (2011). Pathophysiology and current management of pruritus in liver disease. Clinic Res Hepatol Gastroenterol.

[20] Alcon S, Talarmin A, Debruyne M, Falconar A, Deubel V, Flamand M (2002). Enzyme-linked immunosorbent assay specific to Dengue virus type 1 nonstructural protein NS1 reveals circulation of the antigen in the blood during the acute phase of disease in patients experiencing primary or secondary infections. J Clin Microbiol.

[21] Singh MP, Goyal K, Ratho RK (2010). Nonstructural protein NS1: giving a new structure to dengue diagnosis. J Clin Microbiol.

[22] Kumar A, Taksande A, Vilhekar K, Jain M: **Prolonged neonatal cholestasis: a rare manifestation of dengue fever.** *Internet J Pediatr Neonatology* 2007.,**9**(1): Available from: http://ispub.com/IJPN/9/1/3528

